# PS, It’s Complicated: The Roles of Phosphatidylserine and Phosphatidylethanolamine in the Pathogenesis of *Candida albicans* and Other Microbial Pathogens

**DOI:** 10.3390/jof4010028

**Published:** 2018-02-20

**Authors:** Chelsi D. Cassilly, Todd B. Reynolds

**Affiliations:** Department of Microbiology, University of Tennessee, Knoxville, TN 37996, USA; ccassill@vols.utk.edu

**Keywords:** phospholipid, phosphatidylserine, phosphatidylethanolamine, virulence

## Abstract

The phospholipids phosphatidylserine (PS) and phosphatidylethanolamine (PE) play important roles in the virulence of *Candida albicans* and loss of PS synthesis or synthesis of PE from PS (PS decarboxylase) severely compromises virulence in *C. albicans* in a mouse model of systemic candidiasis. This review discusses synthesis of PE and PS in *C. albicans* and mechanisms by which these lipids impact virulence in this fungus. This is further compared to how PS and PE synthesis impact virulence in other fungi, parasites and bacteria. Furthermore, the impact of PS asymmetry on virulence and extracellular vesicle formation in several microbes is reviewed. Finally, the potential for PS and PE synthases as drug targets in these various kingdoms is also examined.

## 1. Introduction

Understanding the roles for lipids in the virulence of microbial pathogens has long been an area of interest. Virulence is a broad area of study, encompassing both host and microbial factors, however, within the last decade the role of microbial physiology in virulence has become more appreciated. Many microbes have complex life cycles or reside in a variety of locations and must sense their environment in order to survive and reproduce. This adjustment to environmental stimuli (e.g., nutrient availability, temperature, pH) plays a large role in the metabolism and virulence of microbes [1,2,3].

Lipids are one of the four main macromolecules (along with nucleic acids, proteins and carbohydrates) essential for cells to function. Depending on their properties, lipids can have many roles in the cell including control of membrane structure and fluidity [4,5], signaling [6], facilitating membrane-associated functions [4,7], virulence [8,9,10,11,12] and drug resistance [4,13,14]. A great deal of research has been conducted to help better understand the role that lipids play in virulence across species and even within strains of the same species [15,16,17].

Within the broad category of lipids are many different subtypes, including sphingolipids, phospholipids and sterols. Nearly all of these have been implicated in virulence across a wide range of pathogens [11,18,19,20,21,22]. Furthermore, some microbes have been shown to have the ability to take up host fatty acids which alter the microbes’ membranes, allowing them to resist antibiotics and other stressors [23,24,25,26]. While there are many reviews describing the general role of lipids in microbial pathogenesis [18,19,27,28,29], this review will focus on a specific subset of aminophospholipids, phosphatidylserine (PS) and phosphatidylethanolamine (PE) and their roles in microbial pathogenesis. PS and PE have been subject to fewer studies than some other phospholipid classes regarding their roles in virulence. However, a number of more recent reports reveal interesting roles for PS and PE in the virulence of *Candida albicans* as well as a variety of protozoan and prokaryotic pathogens. This communication will briefly review PS and PE synthesis and then cover the role of PS and PE as regulators of virulence in *C. albicans.* We will compare this to what has been learned in other eukaryotic pathogens and a few prokaryotes.

## 2. Phosphatidylserine and Phosphatidylethanolamine Synthesis in Microbes

### 2.1. Phosphatidylserine Synthesis Is Similar between Fungi and Bacteria

**Figure 1:**
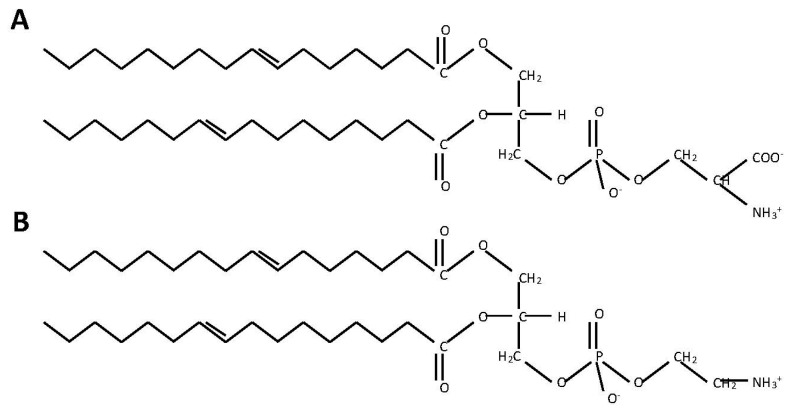
The structure of (**A**) phosphatidylserine and (**B**) phosphatidylethanolamine.

**Figure 2:**
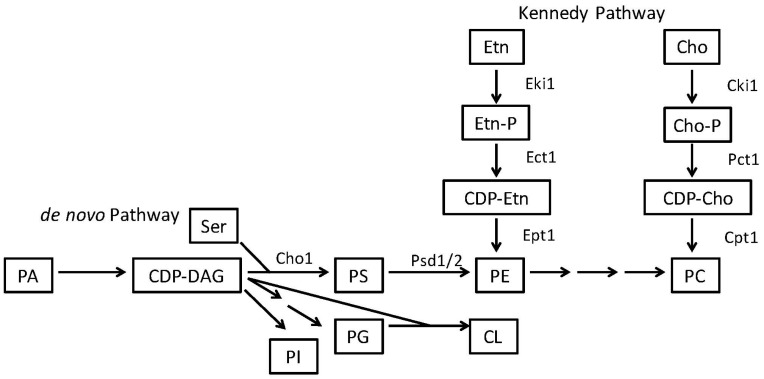
Phospholipid Biosynthesis Pathways in Fungi. Yeasts like *C. albicans* and *S. cerevisiae* synthesize phospholipids via both an endogenous pathway, the *de novo* pathway and an exogenous pathway, the Kennedy pathway. The precursors for producing the most common phospholipids are phosphatidic acid (PA) and CDP-DAG. CDP-DAG is then converted to phosphatidylinositol (PI), PS, or phosphatidylglycerol (PG). The endogenously produced PS can be decarboxylated into PE and then further methylated into PC. In the Kennedy pathway, exogenous ethanolamine (Etn) and/or choline (Cho) are brought into the cell and converted into PE and PC. Abbreviations: CDP-DAG—cytidine diphosphate diacylglycerol; PS—phosphatidylserine; PE—phosphatidylethanolamine; CL—cardiolipin; PC—phosphatidylcholine; Etn—ethanolamine; Cho—choline, Etn-P—phosphoethanolamine, Cho-P—phosphocholine, CDP-Etn—cytidyldiphosphate-ethanolamine, CDP-Cho—cytidyldiphosphatecholine, Ser—serine.

**Figure 3:**
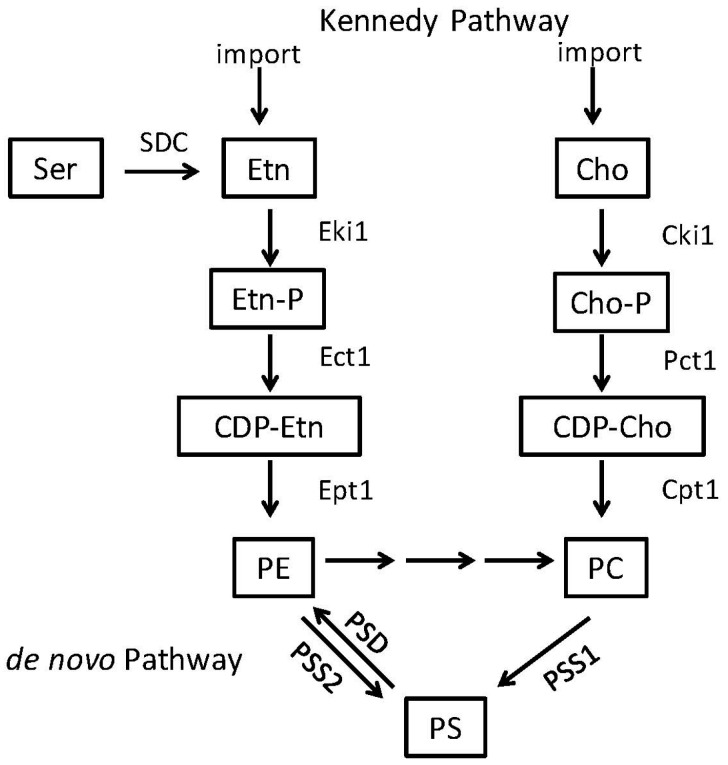
Phospholipid Biosynthesis Pathways in mammals and parasites. Mammals acquire phospholipids via both an endogenous pathway, the *de novo* pathway and a scavenging pathway, the Kennedy pathway. Headgroups of existing PE and PC can be cleaved and replaced with serine to produce PS. PS can be decarboxylated to produce PE. PE can then be methylated three times to produce PC. In the Kennedy pathway, exogenous ethanolamine (Etn) and/or choline (Cho) are brought into the cell and converted into PE and PC. In some parasites, serine (Ser) can be directly decarboxylated to Etn by serine decarboxylase (SDC). Other abbreviations: PS—phosphatidylserine; PE—phosphatidylethanolamine; PC—phosphatidylcholine; Etn—ethanolamine; Cho—choline.

PS is a negatively charged phospholipid with a glycerol backbone and two fatty acid tails (Figure 1A). In bacteria and fungi, PS is produced from two substrates: cytidine diphosphate diacylglycerol (CDP-DAG) and serine (Figure 2). Although the enzymes responsible for this reaction can differ greatly in primary sequence between fungi and many prokaryotes (excepting some bacteria like *Bacillus subtilis* or *Sinorhizobium meliloti* whose PS synthase is similar to *S. cerevisiae*) the mechanism by which they produce PS is similar [4,30,31,32,33,34,35,36]. In mammals and many parasites like *Trypanosoma brucei*, PS is produced through a base-exchange reaction. In mammals, head groups of existing phosphatidylcholine (PC) and PE are cleaved off by two different enzymes, phosphatidylserine synthase-2 (PSS2) and phosphatidylserine synthase-1 (PSS1) respectively and replaced with serine to produce PS [34,37,38,39] (Figure 3).

### 2.2. Phosphatidylethanolamine Is Synthesized by a Variety of Pathways

Phosphatidylethanolamine (PE) is considered a major phospholipid in many eukaryotic organisms and some prokaryotes. It is an uncharged, non-bilayer forming phospholipid due to its small head group, which causes a cone-like shape for its structure (Figure 1B). In eukaryotes and most prokaryotes that contain it, PE is produced by the decarboxylation of PS [4,5,30,40,41]. However, in many eukaryotes PE can alternatively be made from ethanolamine via a scavenging pathway known as the Kennedy Pathway (Figure 2 and Figure 3) [42]. In the Kennedy pathway, ethanolamine is taken up by the cells and phosphorylated to produce phosphoethanolamine, which is then condensed with CTP to produce CDP-ethanolamine. The third and final step is condensation of CDP-ethanolamine with diacylglycerol (DAG) to generate PE and CMP [43].

Although the CDP-DAG and Kennedy pathways are the most common routes employed to generate PE in mammals and fungi, there are alternative mechanisms that produce PE or its precursors in other microbes. In the bacterial plant pathogen *Xanthomonas campestris*, a bifunctional cardiolipin/PE synthase was identified. *X. campestris* produces PE by the decarboxylation of PS; however, upon the deletion of PS decarboxylase, growth of the organism was partially restored when exogenous ethanolamine was supplemented. Researchers identified a putative cardiolipin synthase gene that, in addition to making CL from CDP-DAG and phosphatidylglycerol-phosphate, could produce PE from CDP-DAG and ethanolamine. This process, which may be important in certain conditions, seems to be restricted to Xanthomonadales and Pseudomonadales orders based on phylogenetic analysis [44].

In the kinetoplast parasites such as Trypanomes and *Leishmania*, the Kennedy pathway appears to be the key method of synthesizing PE. In *Trypanosoma brucei* PE is maintained within the cell in two distinct pools [38]. For *Leishmania* most of the ethanolamine used to produce PE is not taken up from the environment but is produced within the organism by cleaving sphingosine-1-phosphate to form long chain fatty aldehydes and phosphoethanolamine. The phosphoethanolamine can be funneled directly into the second step of the Kennedy pathway where phosphoethanolamine is converted to CDP-ethanolamine (Figure 3) [45,46]. This cleavage is carried out by the enzyme sphinosine-1-phosphate lyase (Dpl1), which is also found in other microbes such as *S. cerevisiae*. In yeast, Dpl1 can support growth of yeast in the absence of PS decarboxylase (*psd1∆ psd2∆*) and exogenous ethanolamine, indicating that it can support PE synthesis by the Kennedy pathway in yeast as well. However, under normal conditions, Dpl1 is not a major source for PE synthesis in this organism [47,48,49]. Yeast also have the ability to take up and acylate lyso-PE to produce PE or remodel existing PE species [50,51,52,53,54,55]. *Candida albicans* has a Dpl1 homolog but a role for it in PE synthesis has not been tested.

The apicomplexan parasites also have unusual characteristics regarding PS and PE. The malaria parasite *Plasmodium falciparum* can acquire ethanolamine for the Kennedy pathway by directly decarboxylating serine into ethanolamine, by means of the serine decarboxylase (SDC) enzyme, an enzymatic activity shared with plants but not animals or fungi (Figure 3) [56].

In the intracellular apicomplexan parasite *Toxoplasma gondii*, PE is produced via the Kennedy pathway and via the decarboxylation of PS in the mitochondria [57] as is seen in fungi or mammals. However, *T. gondii* also has unusual versions of the canonical base-exchanging PS synthase and PS decarboxylase enzymes. First, in addition to an internal, membrane-bound PS decarboxylase, it has a second, soluble PS decarboxylase enzyme (*Tg*PSD1) that is secreted extracellularly from *T. gondii* cells and appears to decarboxylate PS to PE in the parasitophorous vacuole, an organelle within the host where *T. gondii* reproduces [57]. This is unusual because both PS decarboxylase and PS synthase are typically membrane bound enzymes with multiple transmembrane domains, although there have been other reports of hyper-expressed PS decarboxylase enzymes dissociating from the cytoplasmic membrane in bacteria [58] and in *Plasmodium falciparum* [59,60]. The function of secreted *Tg*PSD1 within the parasitophorous vacuole is not entirely clear but it could potentially help damage the host cell membrane to allow *T. gondii* to escape the parasitophorous vacuole when it lyses the cell. Furthermore, the secreted enzyme may bind liposomes and host membranes to allow for membrane biogenesis and parasite replication. Third, the secreted enzyme may suppress PS exposure on the apoptotic host cell, thereby avoiding phagocytosis and allowing the parasite to replicate and avoid the immune system. While these are all possible roles, the exact reasons for its unique function still remain to be elucidated [61]. *T. gondii* also appears to have the ability to take up host PE (possibly via a permease) when production of PE is inhibited intracellularly, further increasing the survival and fitness of this organism [57].

## 3. PS and PE Can Act as Modulators of Virulence in *Candida*, Bacteria and Parasites

### 3.1. Candida albicans Requires PE Synthesis from PS to Be Virulent

*Candida albicans* is a commensal fungus that normally inhabits the gut and skin of healthy people. However, immunocompromised individuals are at a high risk of developing bloodstream infections where *C. albicans* can infect the deep organs leading to sepsis [62,63,64]. *C. albicans* is known to produce several virulence factors including hyphae, adhesins, lipases, proteases and the more recently described candidalysin [65,66]. It is also able to hide itself to a limited extent from the innate immune system by a process called masking. Yeast cell walls contain four main components: chitin, mannosylated proteins (mannan), β(1-6)-glucan and β(1-3)-glucan. These components are differentially enriched into two layers, with chitin, β(1-6)-glucan and β(1-3)-glucan in the inner layer. Mannan makes up the majority of the outer surface layer of the cell wall, and β-glucans and chitin are “masked” beneath. β-(1,3)-glucan is a pathogen associate molecular pattern (PAMP) that can be detected by the innate immune receptor Dectin-1 as a signal that the host is infected by a fungal pathogen [67]. Dectin-1 is found on macrophages, dendritic cells, neutrophils and some other immune cells. The proposed layered topology, where mannan masks the immunogenic molecule β(1-3)-glucan is a method of innate immune system evasion by this yeast [21]. Disruption of this layering (i.e., unmasking) makes it easier for the host to recognize β(1,3)-glucan and detect the fungus [68,69,70].

It has been shown that the fungal phosphatidylserine (PS) synthase, Cho1, is absolutely required for virulence of *C. albicans*. In a mouse model of systemic infection, the *cho1∆∆* deletion mutant is unable to cause infection, while mice infected with wild-type or *cho1∆/∆::CHO1* reintegrant strains die within two weeks [71]. In addition, *cho1∆∆* exhibits significant reduction in kidney colonization and is completely cleared from the mice, even when they are made neutropenic with cyclophosphamide [72]. In contrast, mice infected with wild-type *C. albicans* show high kidney burden before succumbing to infection [71].

In addition to a complete loss of PS, the *cho1∆∆* mutation also causes a loss of PE synthesized from PS (Figure 2) [41]. This suggested that the avirulence could be caused by loss of PE as well as PS. A major difference between *cho1∆∆* and *psd1∆∆ psd2∆∆* is that only *cho1∆∆* has increased β(1-3)-glucan unmasking in its cell wall, increasing host immune recognition of this microbe [21]. Thus, other underlying factors related to loss of PE play a role in the loss of virulence but cell wall unmasking driven by the loss of PS may contribute to avirulence in the *cho1∆∆* mutant, as well. The mechanisms responsible for cell wall unmasking in the *cho1∆∆* mutant are currently under investigation.

These defects in virulence in the *cho1∆∆* and *psd1∆∆ psd2∆∆* mutants are manifest despite the presence of an alternative Kennedy pathway for PE synthesis (Figure 2). This brings up questions as to whether Kennedy pathway synthesized PE is able to compensate for PS-derived PE or if cells are unable to make sufficient PE by the Kennedy pathway. Furthermore, this also opens the question of how much of a role the loss of PS alone plays in virulence and whether this impact occurs via unmasking.

**Figure 4:**
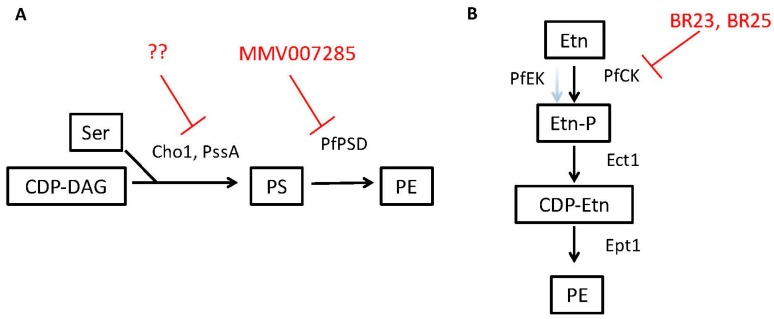
Potential targets and inhibitors for phosphatidylserine and phosphatidylethanolamine synthesis. (**A**) Potential inhibition points for the *de novo* pathway. The phosphatidylserine (PS) synthase from fungi (Cho1) and some bacteria (PssA) that convert cytidine diphosphate diacylglycerol (CDP-DAG) and serine (Ser) to PS would be good candidates for inhibitor development. In addition, the PS decarboxylase enzyme (PSD) that converts PS to phosphatidylethanolamine (PE), which could potentially impact parasite, fungal and select bacterial pathogens is a good point for intervention, and the compound MMV007285 has been reported to inhibit PSD activity in *Plasmodium falciparum*. (**B**) The ethanolamine kinase activity of the *Plasmodium falciparum* choline kinase (PfCK) in the Kennedy pathway can be inhibited by the compounds BR23 and BR25 and appears to be a good point for intervention. Red lines describe points where inhibitors have been discovered (MMV007285 and BR23 and BR25) or are lacking, but would be good points for intervention (??). Other abbreviations: Etn—ethanolamine; Etn-P—phosphoethanolamine, CDP-Etn—cytidyldiphosphate-ethanolamine.

Due to these virulence defects and the reasons that follow, Cho1 represents a good drug target in *C. albicans* (Table 1 and Figure 4A) [71,73]. First, as loss of Cho1 renders *C. albicans* avirulent in mice, inhibition of Cho1 is predicted to render *C. albicans* nonpathogenic in humans. Secondly, since the mammalian PS synthase enzymes are not orthologous with the fungal PS synthase, an inhibitor of Cho1 should be very specific for fungi without affecting mammalian Pss1p and Pss2p (compare Figure 2 and Figure 3). Identification of small molecule inhibitors of Cho1 as potential therapeutics is a priority [73]. Third, Cho1 is conserved throughout pathogenic fungi, so an inhibitor could be broad spectrum [74].

### 3.2. PS Inhibitors Could Be Effective against Some Bacterial Pathogens

Fungal PS synthase inhibitors should be effective against fungal pathogens but they may also be effective against select bacterial pathogens as well. For example, *Brucella abortus* requires PS synthesis for virulence. This bacterium is the causative agent of brucellosis which can be a severe and chronic infection within humans. This organism is a facultative, intracellular pathogen that typically resides within a specific compartment in the host cell called the *Brucella*-containing vacuole (BCV). From this location, *B. abortus* can control the host cell machinery and replicate. Several factors contribute to *B. abortus* virulence including immune modulators, smooth lipopolysaccharide (LPS) and cyclic β-glucans. However, recent studies have shown that the membrane phospholipid composition is crucial for interaction of the microbe with the host cells [75,77]. For example, phosphatidylcholine (PC)—one of the main phospholipids produced in this microbe—is necessary for the organism to set up a chronic infection in a murine model [77,78]. Furthermore, PS and PE play significant roles in virulence as well. A mutant of the phosphatidylserine synthase (*pssA*), was produced, and although PS is not considered a major phospholipid within *B. abortus*, this enzyme is crucial for production of PE (Figure 2) [75,79]. The *pssA* mutant has a loss of PE and shows increased sensitivity to membrane-perturbing agents like SDS. The *pssA* mutant also shows decreased survival intracellularly in tissue culture cells and shows a marked decrease in maturation of the BCV that protects the bacteria intracellularly. Finally, a marked decrease in virulence is found in the *pssA* mutant as compared to the wild-type within a mouse model of infection [75]. Although the exact impact on virulence is not known, it is thought that PE contributes to BCV formation. Furthermore, by disrupting the structure of the membrane, it is possible that important protein complexes or virulence determinants are also disrupted which negatively affects the organism’s ability to survive within host cells.

The *pssA* gene is also important for growth in *Escherichia coli* but it has only been examined for its role in growth in non-pathogenic laboratory strains. Deletion of *pssA* in *E. coli* causes not only a decrease in PS and PE but it also causes a growth defect unless the media is supplemented with divalent cations like Ca^2+^ or Mn^2+^ [80]. Further investigations into the role for PS synthase in the pathogenesis of Gram negative organisms is warranted as it may be important for virulence in a variety of these pathogens (Table 1 and Figure 4A).

### 3.3. PE Synthesis Inhibitors Could Be Effective against Eukaryotic Pathogens

The PS decarboxylase enzymes that convert PS to PE (Psd1 or Psd2) are required for virulence of *C. albicans* as described above, thus these enzymes may be good drug targets in addition to Cho1. However, work on development of eukaryotic PS decarboxylase inhibitors has made greater progress in parasites than in fungi.

*Plasmodium* parasites are the causative agents of malaria, which is one of the most important health problems in the developing world. Finding new treatments with novel modes of actions to better combat this pathogen is a major area of current research because of the rising resistance to existing anti-malarial therapies. The *Plasmodium* PS decarboxylase (PSD) enzyme has been suggested as a drug target since PE is an essential phospholipid in *Plasmodium*. Indeed, inhibition of PSD results in growth arrest of the parasite [59,76,81]. Recent findings have shown that the PSD enzyme from *Plasmodium falciparum*, (found in both soluble and membrane-bound forms in this organism) can complement yeast *psd* mutants [59]. Furthermore, screening a library of known malaria inhibitors identified a particular compound, 7-chloro-*N*-(4-ethoxyphenyl)-4-quinolinamine (MMV007285), with potent activity against *Plasmodium* and the ability to inhibit the catalytic function of PSD (Table 1 and Figure 4A) [59].

In addition to PS decarboxylase, the choline kinase (CK) is crucial for PE synthesis in *Plasmodium* and has been suggested as another drug target [76]. This enzyme is involved in the Kennedy Pathway where choline and ethanolamine are taken up from the environment and used to produce PC and PE, respectively (Figure 3). Recent work has found that known anti-cancer compounds BR23 and BR25 that inhibit human choline kinase cause a dramatic drop in the levels of PE within *P. falciparum* but not PC, as these compounds influence PE synthesis more than PC synthesis in this parasite [76]. The CK of *P. falciparum* is involved in both choline and ethanolamine phosphorylation but the drugs seem to primarily impact ethanolamine phosphorylation, rather than choline phosphorylation, explaining the differential effects on PE and PC synthesis. Treatment with either drug led to arrested development of the parasite, likely as a result of the loss of membrane PE and ultimately were lethal [76]. These findings demonstrate the importance of PE biosynthesis in survival and pathogenicity of some microbes and are some of the first studies where small molecule inhibition of an ethanolamine kinase in a pathogen has led to promising lead inhibitory compounds (Table 1 and Figure 4B).

Finally, although *P. falciparum* has two different pathways to make PE, (both CDP-DAG and Kennedy, Figure 2 and Figure 3), loss of either pathway appears to be sufficient to compromise its growth. This is surprising and indicates several possible explanations: (1) the molecular species of PE made from the two pathways differ and each is crucial for virulence; (2) localization of PE synthesis for each pathway differs (PSD is in the mitochondria while the Kennedy pathway synthesizes PE in the ER) and PE made in one location is not sufficient to make up for the other; (3) the volume of PE made by either pathway alone is not sufficient to support virulence [59,76].

The ability of PS decarboxylase inhibitors to block fungal infections needs to be tested since Psd1 and Psd2 are required for virulence. The effectiveness of choline/ethanolamine kinase inhibitors in this fungus is unclear, as the role of the Kennedy pathway in the virulence of fungi like *Candida albicans* has not yet been reported.

## 4. PS Symmetry in the Membrane Plays a Role in Virulence

The lipid composition in the microbe’s membranes can play a role in promoting virulence but the symmetry of lipids can also play an important role. For example, the symmetry of PS can impact a microbe’s uptake by host cells (e.g., phagocytosis by host macrophages).

### 4.1. Cryptococcus neoformans Lipid Flippase Impacts Virulence

*Cryptococcus neoformans* is a facultative intracellular fungal pathogen that is a leading cause of fungal pathogenesis worldwide [82]. A recent study has demonstrated that Cdc50, a regulatory subunit for lipid flippases that are responsible for maintaining asymmetry in the phospholipid bilayer, is required for virulence [83]. Upon deletion of Cdc50, *C. neoformans* becomes more sensitive to fluconazole, caspofungin and SDS, likely due to a change in membrane integrity. In mice, the *cdc50∆* mutant is unable to cause a robust infection and is cleared from the lungs, further implicating this protein as a virulence factor. The exact mechanism behind this loss of virulence is currently still under investigation. However, PS is normally localized to the inner leaflet of the plasma membrane and is not exposed to the outside of cell, but in the *cdc50∆* mutant there is increased exposure of PS to outside of the membrane. This provides support for the importance of proper PS symmetry for the virulence of this fungus. These results suggest that enzymes needed to maintain PS asymmetry represent good drug targets within *Cryptococcus*. In addition, since the *cdc50∆* mutant showed greater susceptibility to caspofungin, a common antifungal drug that *Cryptococcus* is naturally resistant to, further exploration of phospholipid flippases or membrane asymmetry could improve the effectiveness of echinocandins against the fungus. The role of such flippases remains to be studied in *Candida* spp. and could be an exciting area of study.

### 4.2. PS Exposure in Parasites Facilitates Invasion of Host Cells

Changes in PS symmetry can actually improve virulence in some parasites. *Leishmania braziliensis* is known to have multiple virulence factors associated with disease including cell surface molecules like lipophosphoglycan (LPG) and carbohydrates. In addition, PS also serves as a ligand for mononuclear macrophages. *Leishmania tropica* promastigote forms expose higher levels of PS on their surface during the infective growth phases [84]. Furthermore, amastigotes of *Leishmania amazonensis* with higher levels of PS on the cell surface had increased infectivity in vivo and in vitro [85]. These findings indicate that a higher concentration of PS on the surface of these organisms increases the chances of being internalized by the host macrophages [85]. The PS exposed on the membrane of the parasite is thought to play a role in apoptotic mimicry, allowing *L. brasiliensis* to establish an infection within the host [86,87]. When PS exposed on the surface of *L. brasiliensis*, *Leishmania tropica* or *L. amazonensis* is blocked with annexin V, the infectivity of the parasite in murine peritoneal macrophages is decreased [84,85,86,88,89].

Interestingly, PS exposure seems to have importance even beyond the initial entry into host macrophages as well. In *L. amazonensis* and *L. major*, subpopulations of PS-positive and PS-negative promastigotes cooperate to produce a sustained and successful infection of host macrophages [89,90]. *L. amazonensis* amastigotes with high levels of PS exposed on their cell surfaces are able to induce cytokine production as well as inhibit NO production [85]. These findings implicate PS production and exposure as an excellent drug target within *Leishmania*.

Similar instances of apoptotic mimicry have also been reported for *Trypanosoma cruzi* [91,92], *Toxoplasma gondii* [93] and even enveloped viruses [94,95,96,97], reinforcing the model that PS exposure can regulate infection. Targeting proteins responsible for this PS exposure [98,99] or enzymes involved in PS synthesis, could be a viable option for future therapies across a wide variety of pathogens.

## 5. PS and PE May Play a Role in Extracellular Vesicles in *Candida* and Other Fungi

Another potential contributor to virulence in fungi and other microbes is the use of extracellular vesicles as delivery systems for virulence factors. Extracellular vesicles been observed in *C. albicans*, *Candida parapsilosis*, *Sporothrix schenckii*, *Saccharomyces cerevisiae* [100], *Cryptococcus neoformans* [101,102] and *Paracoccidioides brasiliensis* [103]. The role of extracellular vesicles in parasites and bacteria is more extensively reviewed in [104].

Lipid profiles from 4 different strains of *P. brasiliensis* [103] revealed that the concentration of PC was higher in virulent strains than in the avirulent strain. Further studies into the lipid composition of fungal extracellular vesicles, known to harbor various virulence factors, showed some differences in the lipid composition, which is theorized to play a role in the virulence of different strains of *P. brasiliensis* [105].

*Histoplasma capsulatum* is a pathogenic fungus that can cause life-threatening systemic disease. This organism has many different characteristics that allow it to grow well within the host environment, including survival in a wide pH range and during nutrient starvation. *H. capsulatum* also produces various virulence factors like heat-shock proteins and the cell wall protein YPS3p. Analysis of the composition of extracellular vesicles reveals that the vesicles were made up of common plasma membrane phospholipids including PE, PS and PC [100]. This composition is similar to what is found in mammalian exosomes which are known to transport important molecules like bioactive lipids and lipid-degrading enzymes. The biogenesis of exosomes in mammals is a specific process that requires certain lipids with a characteristic membrane organization [106,107].

In *Candida albicans*, work to determine the effect of altered lipid composition on exosomes has been performed. A recent study found that the *cho1∆∆* mutant of *C. albicans*, which lacks PS in its membrane, displays decreased ability to secrete proteases and phospholipases, and extracellular vesicles with altered protein contents and immune activation properties compared to wild-type. These data indicate the importance of proper phospholipid balance in this process [108]. This has not yet been explored in other fungi using similar genetic approaches, but could indicate that PS is important for extracellular vesicle secretion in these pathogens as well.

## 6. Perspectus

PS and PE play crucial roles in the physiology and pathogenicity of *Candida albicans* but also for a variety of other fungi, parasites, and bacteria. Because of this common theme, we believe that targeting the production of PS and PE, likely by small molecule inhibitors of biosynthetic enzymes, is an area rich with potential for identifying therapeutic drugs [73]. We have covered several possible targets in the course of this review, and they are summarized along with potential lead compounds or drugs in Table 1 and Figure 4. There is also a great deal left to be discovered about which PS and PE pathways impact virulence in these various microbes and how they do so. For instance, PS and PE play crucial roles in the virulence of *Candida albicans* but that has not been explored for the other *Candida* spp. Furthermore, it will be interesting to see how these phospholipid pathways impact virulence in fungi outside of the *Candida* genus. Moreover, the roles they play in parasites and even bacteria may also be informative for studies in fungi. Finally, the inhibitors that are developed for enzymes in one kingdom may serve as starting points for inhibitors of analogous pathways in other kingdoms.

## Figures and Tables

**Table 1:** Potential drug targets among phosphatidylserine and phosphatidylethanolamine synthesis enzymes.

Target/Enzyme Activity	Organism	Phospholipid Product	Inhibitor(s) *	Other Potential Pathogens	Ref.
Cho1/Phosphatidylserine synthase	*Candida albicans*	phosphatidylserine	NA	Conserved in fungi	[71]
PssA/Phosphatidylserine synthase	*Brucella abortus*	phosphatidylserine	NA	Conserved in gram negative bacteria	[75]
PfPSD/Phosphatidylserine decarboxylase	*Plasmodium falciparum*	Phosphatidyl-ethanolamine	7-chloro-*N*-(4-ethoxyphenyl)-4-quinolinamine (MMV007285)	Conserved in parasites, fungi and some bacteria	[59]
PfCK/ Ethanolamine kinase activity of choline kinase	*Plasmodium falciparum*	Phosphatidyl-ethanolamine	BR23, BR25	unknown	[76]

* published molecules that can inhibit enzyme activity; NA-not applicable, as no inhibitors have been reported.

## References

[1] Finlay B.B., Falkow S. (1989). Common themes in microbial pathogenicity. Microbiol. Rev..

[2] Finlay B.B., Falkow S. (1997). Common themes in microbial pathogenicity revisited. Microbiol. Mol. Biol. Rev..

[3] Mahan M.J., Heithoff D.M., Sinsheimer R.L., Low D.A. (2000). Assessment of bacterial pathogenesis by analysis of gene expression in the host. Annu. Rev. Genet..

[4] Cronan J.E. (2003). Bacterial membrane lipids: Where do we stand?. Annu. Rev. Microbiol..

[5] Dowhan W. (1997). Molecular basis for membrane phospholipid diversity: Why are there so many lipids?. Annu. Rev. Biochem..

[6] Shea J.M., Del Poeta M. (2006). Lipid signaling in pathogenic fungi. Curr. Opin. Microbiol..

[7] Bogdanov M., Dowhan W. (1999). Lipid-assisted protein folding. J. Biol. Chem..

[8] Mirucki C.S., Abedi M., Jiang J., Zhu Q., Wang Y.H., Safavi K.E., Clark R.B., Nichols F.C. (2014). Biologic activity of porphyromonas endodontalis complex lipids. J. Endod..

[9] Jain M., Petzold C.J., Schelle M.W., Leavell M.D., Mougous J.D., Bertozzi C.R., Leary J.A., Cox J.S. (2007). Lipidomics reveals control of *Mycobacterium tuberculosis* virulence lipids via metabolic coupling. Proc. Natl. Acad. Sci. USA.

[10] Upreti H.B., Rawat D.S., Das S.K. (1984). Virulence, capsule size and lipid composition interrelation of *Cryptococcus neoformans*. Microbiologica.

[11] Wessel M., Klusener S., Godeke J., Fritz C., Hacker S., Narberhaus F. (2006). Virulence of *Agrobacterium tumefaciens* requires phosphatidylcholine in the bacterial membrane. Mol. Microbiol..

[12] Bhatt A., Molle V., Besra G.S., Jacobs W.R., Kremer L. (2007). The *Mycobacterium tuberculosis* FAS-Ⅱ condensing enzymes: Their role in mycolic acid biosynthesis, acid-fastness, pathogenesis and in future drug development. Mol. Microbiol..

[13] Anderes E.A., Sandine W.E., Elliker P.R. (1971). Lipids of antibiotic-sensitive and -resistant strains of *Pseudomonas aeruginosa*. Can. J. Microbiol..

[14] Rakotomanga M., Saint-Pierre-Chazalet M., Loiseau P.M. (2005). Alteration of fatty acid and sterol metabolism in miltefosine-resistant *Leishmania donovani* promastigotes and consequences for drug-membrane interactions. Antimicrob. Agents Chemother..

[15] Cox R.A., Best G.K. (1972). Cell wall composition of two strains of *Blastomyces dermatitidis* exhibiting differences in virulence for mice. Infect. Immun..

[16] Disalvo A.F., Denton J.F. (1963). Lipid content of four strains of *Blastomyces dermatitidis* of different mouse virulence. J. Bacteriol..

[17] Nielsen H.S. (1965). Variation in lipid content of strains of *Histoplasma capsulatum* exhibiting different virulence properties for mice. J. Bacteriol..

[18] Rella A., Farnoud A.M., Del Poeta M. (2016). Plasma membrane lipids and their role in fungal virulence. Prog. Lipid Res..

[19] Geiger O., Timmis K.N. (2010). Lipids and *Legionella* virulence. Handbook of Hydrocarbon and Lipid Microbiology.

[20] Goren M.B., Brokl O., Schaefer W.B. (1974). Lipids of putative relevance to virulence in *Mycobacterium tuberculosis*: Correlation of virulence with elaboration of sulfatides and strongly acidic lipids. Infect. Immun..

[21] Davis S.E., Hopke A., Minkin S.C., Montedonico A.E., Wheeler R.T., Reynolds T.B. (2014). Masking of β(1-3)-glucan in the cell wall of *Candida albicans* from detection by innate immune cells depends on phosphatidylserine. Infect. Immun..

[22] Tilney L.G., Harb O.S., Connelly P.S., Robinson C.G., Roy C.R. (2001). How the parasitic bacterium *Legionella pneumophila* modifies its phagosome and transforms it into rough ER: Implications for conversion of plasma membrane to the ER membrane. J. Cell Sci..

[23] Giles D.K., Hankins J.V., Guan Z., Trent M.S. (2011). Remodelling of the *Vibrio cholerae* membrane by incorporation of exogenous fatty acids from host and aquatic environments. Mol. Microbiol..

[24] Harp J.R., Saito H.E., Bourdon A.K., Reyes J., Arias C.A., Campagna S.R., Fozo E.M. (2016). Exogenous fatty acids protect *Enterococcus faecalis* from daptomycin-induced membrane stress independently of the response regulator liar. Appl. Environ. Microbiol..

[25] Saito H.E., Harp J.R., Fozo E.M. (2014). Incorporation of exogenous fatty acids protects *Enterococcus faecalis* from membrane-damaging agents. Appl. Environ. Microbiol..

[26] Yao J., Rock C.O. (2017). Exogenous fatty acid metabolism in bacteria. Biochimie.

[27] Sant D.G., Tupe S.G., Ramana C.V., Deshpande M.V. (2016). Fungal cell membrane-promising drug target for antifungal therapy. J. Appl. Microbiol..

[28] Ramakrishnan S., Serricchio M., Striepen B., Butikofer P. (2013). Lipid synthesis in protozoan parasites: A comparison between kinetoplastids and apicomplexans. Prog. Lipid Res..

[29] Mishra P., Bolard J., Prasad R. (1992). Emerging role of lipids of *Candida albicans*, a pathogenic dimorphic yeast. Biochim. Biophys. Acta.

[30] Carman G.M., Han G.S. (2011). Regulation of phospholipid synthesis in the yeast *Saccharomyces cerevisiae*. Annu. Rev. Biochem..

[31] Carman G.M., Zeimetz G.M. (1996). Regulation of phospholipid biosynthesis in the yeast *Saccharomyces cerevisiae*. J. Biol. Chem..

[32] Raetz C.R., Dowhan W. (1990). Biosynthesis and function of phospholipids in *Escherichia coli*. J. Biol. Chem..

[33] Sohlenkamp C., de Rudder K.E., Geiger O. (2004). Phosphatidylethanolamine is not essential for growth of *Sinorhizobium meliloti* on complex culture media. J. Bacteriol..

[34] Vance J.E. (2015). Phospholipid synthesis and transport in mammalian cells. Traffic.

[35] Henry S.A., Kohlwein S.D., Carman G.M. (2012). Metabolism and regulation of glycerolipids in the yeast *Saccharomyces cerevisiae*. Genetics.

[36] Vance J.E., Steenbergen R. (2005). Metabolism and functions of phosphatidylserine. Prog. Lipid Res..

[37] Kuge O., Nishijima M. (1997). Phosphatidylserine synthase I and II of mammalian cells. Biochim. Biophys. Acta.

[38] Signorell A., Rauch M., Jelk J., Ferguson M.A., Butikofer P. (2008). Phosphatidylethanolamine in *Trypanosoma brucei* is organized in two separate pools and is synthesized exclusively by the kennedy pathway. J. Biol. Chem..

[39] Tasseva G., Bai H.D., Davidescu M., Haromy A., Michelakis E., Vance J.E. (2013). Phosphatidylethanolamine deficiency in mammalian mitochondria impairs oxidative phosphorylation and alters mitochondrial morphology. J. Biol. Chem..

[40] Kanfer J., Kennedy E.P. (1964). Metabolism and function of bacterial lipids II. Biosynthesis of phospholipids in *Escherichia coli*. J. Biol. Chem..

[41] Cassilly C.D., Farmer A.T., Montedonico A.E., Smith T.K., Campagna S.R., Reynolds T.B. (2017). Role of phosphatidylserine synthase in shaping the phospholipidome of *Candida albicans*. FEMS Yeast Res..

[42] Kennedy E.P., Weiss S.B. (1956). The function of cytidine coenzymes in the biosynthesis of phospholipides. J. Biol Chem..

[43] Gibellini F., Smith T.K. (2010). The kennedy pathway—*de novo* synthesis of phosphatidylethanolamine and phosphatidylcholine. IUBMB Life.

[44] Moser R., Aktas M., Fritz C., Narberhaus F. (2014). Discovery of a bifunctional cardiolipin/phosphatidylethanolamine synthase in bacteria. Mol. Microbiol..

[45] Pulido S.A., Nguyen V.H., Alzate J.F., Cedeno D.L., Makurath M.A., Rios-Vasquez A., Duque-Benitez S.M., Jones M.A., Robledo S.M., Friesen J.A. (2017). Insights into the phosphatidylcholine and phosphatidylethanolamine biosynthetic pathways in *Leishmania* parasites and characterization of a choline kinase from *Leishmania infantum*. Comp. Biochem. Physiol. B Biochem. Mol. Biol..

[46] Zhang K., Pompey J.M., Hsu F.F., Key P., Bandhuvula P., Saba J.D., Turk J., Beverley S.M. (2007). Redirection of sphingolipid metabolism toward *de novo* synthesis of ethanolamine in *Leishmania*. EMBO J..

[47] Birner R., Burgermeister M., Schneiter R., Daum G. (2001). Roles of phosphatidylethanolamine and of its several biosynthetic pathways in *Saccharomyces cerevisiae*. Mol. Biol. Cell..

[48] Mao C., Wadleigh M., Jenkins G.M., Hannun Y.A., Obeid L.M. (1997). Identification and characterization of *Saccharomyces cerevisiae* dihydrosphingosine-1-phosphate phosphatase. J. Biol. Chem..

[49] Saba J.D., Nara F., Bielawska A., Garrett S., Hannun Y.A. (1997). The *BST1* gene of *Saccharomyces cerevisiae* is the sphingosine-1-phosphate lyase. J. Biol. Chem..

[50] Deng L., Fukuda R., Kakihara T., Narita K., Ohta A. (2010). Incorporation and remodeling of phosphatidylethanolamine containing short acyl residues in yeast. Biochim. Biophys. Acta.

[51] Flis V.V., Daum G. (2013). Lipid transport between the endoplasmic reticulum and mitochondria. Cold Spring Harb. Perspect. Biol..

[52] Riekhof W.R., Voelker D.R. (2006). Uptake and utilization of lyso-phosphatidylethanolamine by *Saccharomyces cerevisiae*. J. Biol. Chem..

[53] Riekhof W.R., Wu J., Gijon M.A., Zarini S., Murphy R.C., Voelker D.R. (2007). Lysophosphatidylcholine metabolism in *Saccharomyces cerevisiae*: The role of P-type ATPases in transport and a broad specificity acyltransferase in acylation. J. Biol. Chem..

[54] Riekhof W.R., Wu J., Jones J.L., Voelker D.R. (2007). Identification and characterization of the major lysophosphatidylethanolamine acyltransferase in *Saccharomyces cerevisiae*. J. Biol. Chem..

[55] Burgermeister M., Birner-Grunberger R., Nebauer R., Daum G. (2004). Contribution of different pathways to the supply of phosphatidylethanolamine and phosphatidylcholine to mitochondrial membranes of the yeast *Saccharomyces cerevisiae*. Biochim. Biophys. Acta.

[56] Elabbadi N., Ancelin M.L., Vial H.J. (1997). Phospholipid metabolism of serine in plasmodium-infected erythrocytes involves phosphatidylserine and direct serine decarboxylation. Biochem. J..

[57] Hartmann A., Hellmund M., Lucius R., Voelker D.R., Gupta N. (2014). Phosphatidylethanolamine synthesis in the parasite mitochondrion is required for efficient growth but dispensable for survival of *Toxoplasma gondii*. J. Biol. Chem..

[58] Tyhach R.J., Hawrot E., Satre M., Kennedy E.P. (1979). Increased synthesis of phosphatidylserine decarboxylase in a strain of *Escherichia coli* bearing a hybrid plasmid. Altered association of enzyme with the membrane. J. Biol. Chem..

[59] Choi J.Y., Kumar V., Pachikara N., Garg A., Lawres L., Toh J.Y., Voelker D.R., Ben Mamoun C. (2016). Characterization of plasmodium phosphatidylserine decarboxylase expressed in yeast and application for inhibitor screening. Mol. Microbiol..

[60] Baunaure F., Eldin P., Cathiard A.M., Vial H. (2004). Characterization of a non-mitochondrial type I phosphatidylserine decarboxylase in *Plasmodium falciparum*. Mol. Microbiol..

[61] Gupta N., Hartmann A., Lucius R., Voelker D.R. (2012). The obligate intracellular parasite *Toxoplasma gondii* secretes a soluble phosphatidylserine decarboxylase. J. Biol. Chem..

[62] Bustamante C.I. (2005). Treatment of *Candida* infection: A view from the trenches!. Curr. Opin. Infect. Dis..

[63] Eggimann P., Garbino J., Pittet D. (2003). Management of *Candida* species infections in critically ill patients. Lancet Infect. Dis..

[64] Cassone A., Cauda R. (2012). *Candida* and candidiasis in hiv-infected patients: Where commensalism, opportunistic behavior and frank pathogenicity lose their borders. Aids.

[65] Moyes D.L., Wilson D., Richardson J.P., Mogavero S., Tang S.X., Wernecke J., Hofs S., Gratacap R.L., Robbins J., Runglall M. (2016). Candidalysin is a fungal peptide toxin critical for mucosal infection. Nature.

[66] Kumamoto C.A., Vinces M.D. (2005). Contributions of hyphae and hypha-co-regulated genes to *Candida albicans* virulence. Cell. Microbiol..

[67] Brown G.D., Gordon S. (2001). Immune recognition. A new receptor for β-glucans. Nature.

[68] Wheeler R.T., Fink G.R. (2006). A drug-sensitive genetic network masks fungi from the immune system. PLoS Pathog..

[69] Wheeler R.T., Kombe D., Agarwala S.D., Fink G.R. (2008). Dynamic, morphotype-specific *Candida albicans* β-glucan exposure during infection and drug treatment. PLoS Pathog..

[70] Hasim S., Allison D.P., Retterer S.T., Hopke A., Wheeler R.T., Doktycz M.J., Reynolds T.B. (2017). β-(1,3)-glucan unmasking in some *Candida albicans* mutants correlates with increases in cell wall surface roughness and decreases in cell wall elasticity. Infect. Immun..

[71] Chen Y.L., Montedonico A.E., Kauffman S., Dunlap J.R., Menn F.M., Reynolds T.B. (2010). Phosphatidylserine synthase and phosphatidylserine decarboxylase are essential for cell wall integrity and virulence in *Candida albicans*. Mol. Microbiol..

[72] Chen Y.L., Reynolds T.B. (2009).

[73] Cassilly C.D., Maddox M.M., Cherian P.T., Bowling J.J., Hamann M.T., Lee R.E., Reynolds T.B. (2016). SB-224289 antagonizes the antifungal mechanism of the marine depsipeptide papuamide A. PLoS ONE.

[74] Braun B.R., van Het Hoog M., d’Enfert C., Martchenko M., Dungan J., Kuo A., Inglis D.O., Uhl M.A., Hogues H., Berriman M. (2005). A human-curated annotation of the *Candida albicans* genome. PLoS Genet..

[75] Bukata L., Altabe S., de Mendoza D., Ugalde R.A., Comerci D.J. (2008). Phosphatidylethanolamine synthesis is required for optimal virulence of *Brucella abortus*. J. Bacteriol..

[76] Serran-Aguilera L., Denton H., Rubio-Ruiz B., Lopez-Gutierrez B., Entrena A., Izquierdo L., Smith T.K., Conejo-Garcia A., Hurtado-Guerrero R. (2016). *Plasmodium falciparum* choline kinase inhibition leads to a major decrease in phosphatidylethanolamine causing parasite death. Sci. Rep..

[77] Comerci D.J., Altabe S., de Mendoza D., Ugalde R.A. (2006). *Brucella abortus* synthesizes phosphatidylcholine from choline provided by the host. J. Bacteriol..

[78] Conde-Alvarez R., Grillo M.J., Salcedo S.P., de Miguel M.J., Fugier E., Gorvel J.P., Moriyon I., Iriarte M. (2006). Synthesis of phosphatidylcholine, a typical eukaryotic phospholipid, is necessary for full virulence of the intracellular bacterial parasite *Brucella abortus*. Cell. Microbiol..

[79] Thiele O.W., Kehr W. (1969). Die “freien” lipids of *Brucella abortus* bang. FEBS J..

[80] Nakajima M., DeChavigny A., Johnson C.E., Hamada J., Stein C.A., Nicolson G.L. (1991). Suramin. A potent inhibitor of melanoma heparanase and invasion. J. Biol. Chem..

[81] Ben Mamoun C., Prigge S.T., Vial H. (2010). Targeting the lipid metabolic pathways for the treatment of malaria. Drug Dev. Res..

[82] Brown G.D., Denning D.W., Gow N.A., Levitz S.M., Netea M.G., White T.C. (2012). Hidden killers: Human fungal infections. Sci. Transl. Med..

[83] Huang W., Liao G., Baker G.M., Wang Y., Lau R., Paderu P., Perlin D.S., Xue C. (2016). Lipid flippase subunit cdc50 mediates drug resistance and virulence in *Cryptococcus neoformans*. MBio.

[84] Tripathi A., Gupta C.M. (2003). Transbilayer translocation of membrane phosphatidylserine and its role in macrophage invasion in *Leishmania* promastigotes. Mol. Biochem. Parasitol..

[85] Wanderley J.L., Moreira M.E., Benjamin A., Bonomo A.C., Barcinski M.A. (2006). Mimicry of apoptotic cells by exposing phosphatidylserine participates in the establishment of amastigotes of *Leishmania* (l) amazonensis in mammalian hosts. J. Immunol..

[86] De Freitas Balanco J.M., Moreira M.E., Bonomo A., Bozza P.T., Amarante-Mendes G., Pirmez C., Barcinski M.A. (2001). Apoptotic mimicry by an obligate intracellular parasite downregulates macrophage microbicidal activity. Curr. Biol..

[87] Van Zandbergen G., Solbach W., Laskay T. (2007). Apoptosis driven infection. Autoimmunity.

[88] Farias L.H., Rodrigues A.P., Silveira F.T., Seabra S.H., DaMatta R.A., Saraiva E.M., Silva E.O. (2013). Phosphatidylserine exposure and surface sugars in two *Leishmania* (viannia) braziliensis strains involved in cutaneous and mucocutaneous leishmaniasis. J. Infect. Dis..

[89] Wanderley J.L., Pinto da Silva L.H., Deolindo P., Soong L., Borges V.M., Prates D.B., de Souza A.P., Barral A., Balanco J.M., do Nascimento M.T. (2009). Cooperation between apoptotic and viable metacyclics enhances the pathogenesis of leishmaniasis. PLoS ONE.

[90] Van Zandbergen G., Bollinger A., Wenzel A., Kamhawi S., Voll R., Klinger M., Muller A., Holscher C., Herrmann M., Sacks D. (2006). *Leishmania* disease development depends on the presence of apoptotic promastigotes in the virulent inoculum. Proc. Natl. Acad. Sci. USA.

[91] Damatta R.A., Seabra S.H., Deolindo P., Arnholdt A.C., Manhaes L., Goldenberg S., de Souza W. (2007). *Trypanosoma cruzi* exposes phosphatidylserine as an evasion mechanism. FEMS Microbiol. Lett..

[92] Freire-de-Lima C.G., Nascimento D.O., Soares M.B., Bozza P.T., Castro-Faria-Neto H.C., de Mello F.G., DosReis G.A., Lopes M.F. (2000). Uptake of apoptotic cells drives the growth of a pathogenic trypanosome in macrophages. Nature.

[93] Seabra S.H., de Souza W., Damatta R.A. (2004). *Toxoplasma gondii* exposes phosphatidylserine inducing a TGF-β_1_ autocrine effect orchestrating macrophage evasion. Biochem. Biophys. Res. Commun..

[94] Moller-Tank S., Kondratowicz A.S., Davey R.A., Rennert P.D., Maury W. (2013). Role of the phosphatidylserine receptor TIM-1 in enveloped-virus entry. J. Virol..

[95] Jemielity S., Wang J.J., Chan Y.K., Ahmed A.A., Li W., Monahan S., Bu X., Farzan M., Freeman G.J., Umetsu D.T. (2013). TIM-family proteins promote infection of multiple enveloped viruses through virion-associated phosphatidylserine. PLoS Pathog..

[96] Soares M.M., King S.W., Thorpe P.E. (2008). Targeting inside-out phosphatidylserine as a therapeutic strategy for viral diseases. Nat. Med..

[97] Vanlandschoot P., Leroux-Roels G. (2003). Viral apoptotic mimicry: An immune evasion strategy developed by the hepatitis b virus?. Trends Immunol..

[98] Dos Santos M.G., Muxel S.M., Zampieri R.A., Pomorski T.G., Floeter-Winter L.M. (2013). Transbilayer dynamics of phospholipids in the plasma membrane of the *Leishmania* genus. PLoS ONE.

[99] Araujo-Santos J.M., Gamarro F., Castanys S., Herrmann A., Pomorski T. (2003). Rapid transport of phospholipids across the plasma membrane of *Leishmania infantum*. Biochem. Biophys. Res. Commun..

[100] Albuquerque P.C., Nakayasu E.S., Rodrigues M.L., Frases S., Casadevall A., Zancope-Oliveira R.M., Almeida I.C., Nosanchuk J.D. (2008). Vesicular transport in *Histoplasma capsulatum*: An effective mechanism for trans-cell wall transfer of proteins and lipids in ascomycetes. Cell. Microbiol..

[101] Rodrigues M.L., Nakayasu E.S., Oliveira D.L., Nimrichter L., Nosanchuk J.D., Almeida I.C., Casadevall A. (2008). Extracellular vesicles produced by *Cryptococcus neoformans* contain protein components associated with virulence. Eukaryot. Cell.

[102] Oliveira D.L., Freire-de-Lima C.G., Nosanchuk J.D., Casadevall A., Rodrigues M.L., Nimrichter L. (2010). Extracellular vesicles from *Cryptococcus neoformans* modulate macrophage functions. Infect. Immun..

[103] Manocha M.S., San-Blas G., Centeno S. (1980). Lipid composition of *Paracoccidioides brasiliensis*: Possible correlation with virulence of different strains. J. Gen. Microbiol..

[104] Schorey J.S., Cheng Y., Singh P.P., Smith V.L. (2015). Exosomes and other extracellular vesicles in host-pathogen interactions. EMBO Rep..

[105] Vallejo M.C., Nakayasu E.S., Longo L.V., Ganiko L., Lopes F.G., Matsuo A.L., Almeida I.C., Puccia R. (2012). Lipidomic analysis of extracellular vesicles from the pathogenic phase of *Paracoccidioides brasiliensis*. PLoS ONE.

[106] Laulagnier K., Motta C., Hamdi S., Roy S., Fauvelle F., Pageaux J.F., Kobayashi T., Salles J.P., Perret B., Bonnerot C. (2004). Mast cell- and dendritic cell-derived exosomes display a specific lipid composition and an unusual membrane organization. Biochem. J..

[107] Subra C., Laulagnier K., Perret B., Record M. (2007). Exosome lipidomics unravels lipid sorting at the level of multivesicular bodies. Biochimie.

[108] Wolf J.M., Espadas J., Luque-Garcia J., Reynolds T., Casadevall A. (2015). Lipid biosynthetic genes affect *Candida albicans* extracellular vesicle morphology, cargo, and immunostimulatory properties. Eukaryot. Cell.

